# Assessing the contribution of wind and water erosion in the agro-pastoral ecotone of Northern China with ^137^Cs tracer technology

**DOI:** 10.1038/s41598-025-99457-z

**Published:** 2025-04-27

**Authors:** Hongtao Jiang, Chunrong Guo, Xiaojia Li, Wanfeng Zhang, Pengfei Du, Qiankun Guo, Yousheng Wang, Jing Wang

**Affiliations:** 1 College of Geographical Science, Inner Mongolia Normal University, Hohhot, 010022 Inner Mongolia China; 2 Inner Mongolia Autonomous Region Land Use and Renovation Engineering Technology Research Center, Hohhot, 010022 Inner Mongolia China; 3 Key Laboratory of Mongolian Plateau’s Climate System at Universities of Inner Mongolia Autonomous Region, Hohhot, 010022 Inner Mongolia China; 4 Inner Mongolia Territorial and Space Planning Institute, Hohhot Inner Mongolia, Hohhot, 010013 Inner Mongolia China; 5 China Institute of Water Resources and Hydropower Research, Beijing, 100048 PR China; 6 Inner Mongolia University of Finance and Economics School of Resources and Environmental Economics, Hohhot, 010070 Inner Mongolia China

**Keywords:** Environmental impact, Natural hazards

## Abstract

This study addresses the critical ecological challenges of soil wind and water erosion in the agro-pastoral ecotone of northern China, both of which significantly contribute to soil degradation. Understanding the relative contributions of these erosion types is essential for developing effective control measures. Using the^136^Cs tracer method, we quantified the ratio of soil wind erosion to water erosion under varying topographic and geomorphic conditions. The results revealed that cropland has experienced the most severe erosion in recent decades. Specifically, on gentle slopes (6°–8°), the rate of water erosion exceeded wind erosion by approximately eightfold. On steeper slopes (10°–15°), this trend was even more pronounced, with water erosion surpassing wind erosion by a factor of approximately 27. These findings were corroborated by measured data from a previous study area. Overall, water erosion is the dominant process in the agro-pastoral ecotone of northern China, with wind erosion playing a secondary role. Future erosion prevention strategies should prioritize hydraulic erosion control measures, particularly on sloping cropland. Furthermore, advancing research on the compound mechanisms of wind and water erosion is imperative for developing integrated mitigation strategies, ultimately supporting the sustainable development of the region’s ecological environment.

## Introduction

The agro-pastoral ecotone of northern China lies within the transitional belt between the eastern monsoon climate region and the arid to semi-arid areas in the northwest^1^. This region’s agriculture and animal husbandry are shaped by the combined influences of natural processes and human activities, resulting in a dynamic and narrow landscape pattern. Land use practices here integrate both farming and grazing, with a prevalent coexistence of wind and water erosion, making it a representative example of composite erosion zones^2^.

In particular, the area at the intersection of Shanxi, Shaanxi, and Inner Mongolia, located at the core of the Loess Plateau, exhibits the highest erosion modulus and sand content. This region is one of the primary contributors to sediment load in the Yellow River^3,4^. The influx of this sediment not only exacerbates the pressure on agricultural irrigation and water supply in the Yellow River Basin but also hampers sediment transport in river channels, leading to increased sediment deposition and the intensification of flood disasters.

Furthermore, the agro-pastoral ecotone makes significant contributions to wind and sand sources affecting Beijing, Tianjin, and Hebei^5–9^. According to statistical data, over 50% of moderate to severe air pollution events in the Beijing-Tianjin-Hebei region during the winter and spring seasons are associated with sandstorm weather^10^. The sediment and windborne sand transported from these agro-pastoral ecotone pose severe threats to the ecological stability and societal well-being of both the Yellow River Basin and the Beijing-Tianjin-Hebei region. In recent years, soil erosion in this area has become increasingly severe, with the erosion rate significantly exceeding the national average^11^. Along with rapid population growth and unsustainable land use practices, land degradation has become an increasingly prominent issue^12^. Therefore, it is imperative to quantitatively assess the rates of wind and water erosion in this region and implement effective control measures.

The study of wind-water composite erosion began in the early 20th century, initially focusing on debates regarding the independence of wind and water erosion, gradually evolving into an integrated understanding of their interactions. From the early to mid-20th century, scholars generally considered wind erosion and water erosion as two independent processes, with research primarily centered on exploring the relative dominance of these two forms of erosion^13^^–^^15^. However, by the 1930s to 1950s, researchers gradually recognized the significance of wind-water interactions. Through field investigations in desert regions, researchers from Germany and the Soviet Union revealed the combined influence of alternating aeolian and fluvial processes on geomorphological formation^16,17^. Their studies found that the Potohar Plateau and its surrounding areas experienced the most severe water erosion, while the Kharan and Thar desert regions were dominated by wind erosion. Additionally, the Sulaiman and Kirthar mountain ranges exhibited typical characteristics of wind-water composite erosion^18^.

Since the 1980s, significant progress has been made in the study of wind-water composite erosion, driven by the deepening development of erosion theory, advancements in remote sensing, GIS technologies, and tracer methodologies. These technological innovations, particularly those facilitated through international collaboration, have greatly advanced research into wind-water composite erosion, yielding remarkable achievements, especially in regions such as the Loess Plateau in China. Studies have shown that between 2000 and 2015, the intensity of wind and water erosion decreased significantly, primarily due to the synergistic effects of government-led ecological restoration projects, increased precipitation, and reduced wind speeds^19^.

Wind and water erosion are not independent processes but form a complex coupled erosion mechanism. As noted by Kocurek^20^, the interaction between wind and water erosion is primarily reflected in the transfer of material and energy. Brunsden^21^ proposed the concepts of “decoupled, tightly coupled, and over-coupled” states to describe the relationships between these two erosion processes under varying environmental conditions: in the decoupled state, wind and water erosion operate independently; in the tightly coupled state, energy and material freely flow between the two; while in the over-coupled state, environmental changes weaken the interaction between them. During specific seasonal conditions, the dominant role of wind or water erosion may shift^22,23^.

The impact of composite wind-water erosion on the soil environment is significant, and this process is exacerbated by overgrazing and unsustainable farming practices. In particular, in the northern agro-pastoral ecotone, such human activities have accelerated the alternating effects of wind and water erosion^24^. In summary, the complexity of wind-water erosion processes, along with their spatial and temporal variability, greatly increases the difficulty of accurately reconstructing erosion processes. Consequently, many scholars have focused on analyzing erosion rates to uncover the intrinsic mechanisms of composite erosion. Accurate quantification of erosion rates not only enhances our understanding of the dynamics of erosion processes but also provides critical insights for optimizing land use and formulating effective prevention and control strategies.

Currently, the study of wind-water composite erosion rates primarily relies on several core methodologies, including mathematical modeling, remote sensing and GIS technologies, erosion kinetic analysis, radioactive tracers, and field observations^25^^–^^33^.

Mathematical modeling, as a crucial tool in wind-water composite erosion research, dates back to 1948 when Smith and Whitt first proposed the soil loss equation. This equation integrated variables such as soil loss, slope gradient, slope length, soil erodibility, and soil conservation measures, and it was successfully applied in practical research^25^. Subsequently, in 1965, Wischmeier further expanded this model and introduced the Universal Soil Loss Equation (USLE), which comprehensively encompassed various factors influencing soil erosion. Due to its simplicity and relatively accurate results, the USLE model remains an essential tool for soil erosion assessment and soil conservation planning^26^.

In recent years, researchers worldwide have optimized and adapted the USLE model to suit the specific characteristics of different regions. For instance, China developed the Chinese Soil Loss Equation (CSLE), which is tailored to the country’s diverse topography and climatic conditions^27^. In the field of wind erosion research, the Wind Erosion Equation (WEQ) and its revised version, the Revised Wind Erosion Equation (RWEQ), have been widely applied in the United States and other regions since the 1980s. These models are favored for their flexibility and ease of computation; however, their accuracy heavily depends on the quality of input data and the precision of observational data^29,30^.

To enhance the applicability of models, many studies have integrated simulation models with empirical data. For example, by combining the USLE model with ^136^Cs tracer techniques, it is possible to quantitatively evaluate the composite effects of wind and water erosion^24,28^. However, the accuracy of these models remains constrained by the precision of input variables, particularly the P factor (the soil conservation practices factor), which often requires extensive experimental data for precise evaluation. Furthermore, the spatial variability of climate, environment, and topography can lead to deviations in model outcomes^28^.

With the continuous advancement of remote sensing and GIS technologies, these tools have been widely applied in soil erosion research, particularly in the application and evaluation of models, significantly improving research efficiency and accuracy^34^^–^^36^. Remote sensing technology efficiently extracts key information, such as vegetation cover, soil moisture, and surface roughness, through multispectral and hyperspectral imagery. This information directly supports the evaluation of the C factor (cover management factor) and K factor (soil erodibility factor) in the USLE model^32,37^^–^^40^. GIS technology demonstrates unique advantages in terrain parameter extraction, spatial analysis, and information integration. For instance, in the USLE model, GIS leverages Digital Elevation Model (DEM) data to accurately extract slope steepness (S factor) and slope length (L factor), while effectively facilitating the spatial representation of rainfall erosivity (R factor). For wind erosion models such as RWEQ and WEPS, GIS’s capabilities in spatial interpolation and overlay analysis aid in accurately calculating surface factors, making it an essential tool for multi-factor comprehensive assessments^34,36,41^^–^^43^.

However, the accuracy of remote sensing and GIS methods is, to some extent, constrained by data resolution and quality. For instance, the resolution of DEM data directly affects the precision of slope length and slope steepness factor extraction. In regions with high spatial heterogeneity, GIS interpolation may introduce errors, thereby reducing the capacity to accurately analyze non-homogeneous surface geographic factors. Consequently, future research should prioritize data integration and algorithm optimization to enhance the applicability and predictive accuracy of models^44^.

Furthermore, the incorporation of field observation verification remains a critical step to ensure the reliability of model applications, particularly in soil erosion studies involving complex terrains or significant multi-factor interactions. In current studies on wind-water composite erosion rates, scholars have explored the interaction mechanisms between wind and water erosion processes from the perspective of erosion dynamics^45^^–^^47^. Research indicates that wind and water erosion are not solely driven by individual factors but are influenced by a combination of variables, including climate, topography, soil properties, and vegetation cover^22,48,49^. These factors collectively determine the processes of soil particle detachment, transportation, and deposition under varying conditions.

In theoretical modeling, the rainfall erosivity factor in the USLE model and the wind erosion factor in the RWEQ model are widely used to calculate erosion dynamics and serve as critical bases for predicting wind-water composite erosion rates^50,51^. For instance, Yang successfully predicted wind and water erosion rates using kinetic energy factors^22^. However, wind energy and rainfall energy do not exist in isolation under natural conditions but frequently interact during composite erosion processes, significantly influencing the overall erosion rate. Helming further pointed out that during extreme weather events (e.g., typhoons)^52^, wind forces can markedly alter the distribution of rainfall energy on slopes, thereby affecting rainfall erosivity and its overall contribution to erosion rates. Thus, when evaluating wind-water composite erosion rates, it is essential to fully account for the complex effects of wind-rain interactions on erosion processes.

Radioactive tracer methods are vital tools in soil erosion research, with ¹³⁷Cs tracer technology being a prominent example. Due to its advantages of not requiring continuous monitoring, fast processing, simplicity, and high precision, it has been widely applied in soil erosion studies^53^. ¹³⁷Cs is an artificial radionuclide primarily originating from 20th-century nuclear tests and nuclear accidents, with a half-life of 30.17 years. Once released into the atmosphere, ¹³⁷Cs is deposited onto the surface through dry and wet deposition and is rapidly and strongly adsorbed by clay minerals and organic matter in the soil. The amounts absorbed by plants or lost through leaching processes are extremely limited^54^^–^^58^. This characteristic ensures that the redistribution of ¹³⁷Cs in soil is primarily associated with the mechanical movement of soil particles, and its distribution changes can directly reflect soil erosion or deposition processes. Additionally, some studies have combined the ¹³⁷Cs tracer method with other approaches to quantify the relative contributions of wind and water erosion, providing reliable data to support erosion research^59,60^.

Currently, the ^136^Cs tracer method has been widely applied in the study of soil wind erosion^61,62^ and water erosion^61^^–^^66^, particularly demonstrating significant advantages in estimating soil erosion rates on multi-year average scales. This method provides critical scientific support for soil erosion control and management and has been widely recognized by scholars both domestically and internationally. For instance, Houshia analyzed the erosion and deposition processes of agricultural soils in the Al-Yamoun region of Palestine, finding that soil erosion rates are influenced by multiple factors, including the alternating effects of wind and water erosion^67^. Similarly, Zhang using data from 1963 to 2021, estimated wind erosion in the grasslands of Inner Mongolia, China, and pointed out that while the Revised Wind Erosion Equation (RWEQ) accurately predicts wind erosion rates for croplands, it may overestimate wind erosion rates for grasslands^68^. Additionally, Jiang employed the^136^Cs tracer method to study wind erosion in the black soil region of northeastern China, revealing significant wind erosion rates and further elucidating the relationship between wind erosion and soil depth^69^.

Although the^136^Cs tracer method has significant potential for widespread application, its accuracy is constrained by the stability of background values and the precision of field data validation^70,71^. Background values, referring to the natural levels of^136^Cs in undisturbed soils^72^, are critical for ensuring reliable estimations of erosion rates, as overestimated or underestimated values can significantly affect the evaluation results of wind or water erosion^73^. Therefore, reasonable adjustments and calibrations of background values are necessary during research to ensure that the measurement data accurately reflect the actual soil erosion conditions. For example, in studies conducted on the Qinghai-Tibet Plateau, background values may be influenced by factors such as altitude and soil type, requiring precise measurement to ensure assessment accuracy^74,75^. Similarly, in the northeastern black soil region, the accuracy of background values plays a decisive role in the calculation of water and wind erosion rates^76^.

On the other hand, the validity and operability of field data validation are crucial for ensuring the scientific accuracy of estimation results. Since the^136^Cs tracer method relies on a series of assumptions and model parameters, field data can not only verify the rationality of these assumptions but also identify potential spatial and temporal biases. By comparing the method with other techniques, such as sand collectors or^7^ Be tracer technology, model parameters can be further optimized, thereby improving the scientific accuracy of estimation results^77^. For instance, in certain wind erosion studies on the Loess Plateau, wind erosion rates measured using^136^Cs have shown a high degree of consistency with results obtained from other techniques, such as the BSNE sand collector and^7^ Be technology^78^. This consistency indicates that the method exhibits good reliability under different environmental conditions.

Field observations are the traditional method for studying soil wind and water erosion. By installing instruments in the field, data on particle movement and soil loss during wind and water erosion can be collected. The first systematic observations of wind erosion were conducted by Bagnold in the Libyan desert in the 1940s^14^. In the same year, Chepil applied Bagnold’s wind-sand collector to study wind erosion in agricultural fields^79^. Building on this foundation, Fryrear and colleagues developed the WEQ wind erosion model for the U.S. Great Plains, facilitating field validation and further refinement of models such as WEPS and RWEQ^80,81^. Internationally, the MWAC and BSNE sand traps are widely used for wind erosion observations. The MWAC, designed by Wilson and Cooke in 1980, enables long-term measurements of leptokurtic erosion and can be adapted to diverse terrain conditions; it was later improved by Goossens^82^. The BSNE sand trap, designed by Fryrear in 1998, is adjustable to wind direction and suitable for monitoring the horizontal migration of wind-eroded particles. Wind erosion is calculated based on the difference in sand transport between two traps aligned with the prevailing wind^83,84^.

Additionally, the runoff plot method is a classic approach to quantifying water erosion. This method has been used in parts of the United States since the 1930s^85,86^ to measure water-induced soil loss on slopes directly by controlling soil properties, slope, and vegetation within the plot. The method was later standardized by Renard et al., providing critical data for the parameterization of the USLE model^87^. In summary, field observation methods supply empirical data through direct measurements of wind and water erosion, which are vital for validating and refining erosion models. These techniques offer valuable data for understanding erosion mechanisms and form a scientific basis for developing soil conservation and erosion control strategies.

In summary, this study integrated wind and water erosion research methodologies and utilized the^136^Cs tracer technique to assess wind and water erosion rates across different land use types in the agro-pastoral ecotone of northern China. The water erosion data were validated using the runoff plot method. Similarly, wind erosion rates were validated with BSNE (Big Spring Number Eight) agro-pastoral ecotone sand collector data from adjacent regions. This study quantitatively analyzed the relative contributions of wind and water erosion, providing a scientific basis for understanding the soil erosion characteristics in the agro-pastoral ecotone. Additionally, the findings offer valuable data support for designing soil and water conservation measures tailored to the region’s environmental characteristics. These results contribute to the optimization of land use planning and sustainable development in the area.

## Materials and methods

### Study area

**Fig. 1:**
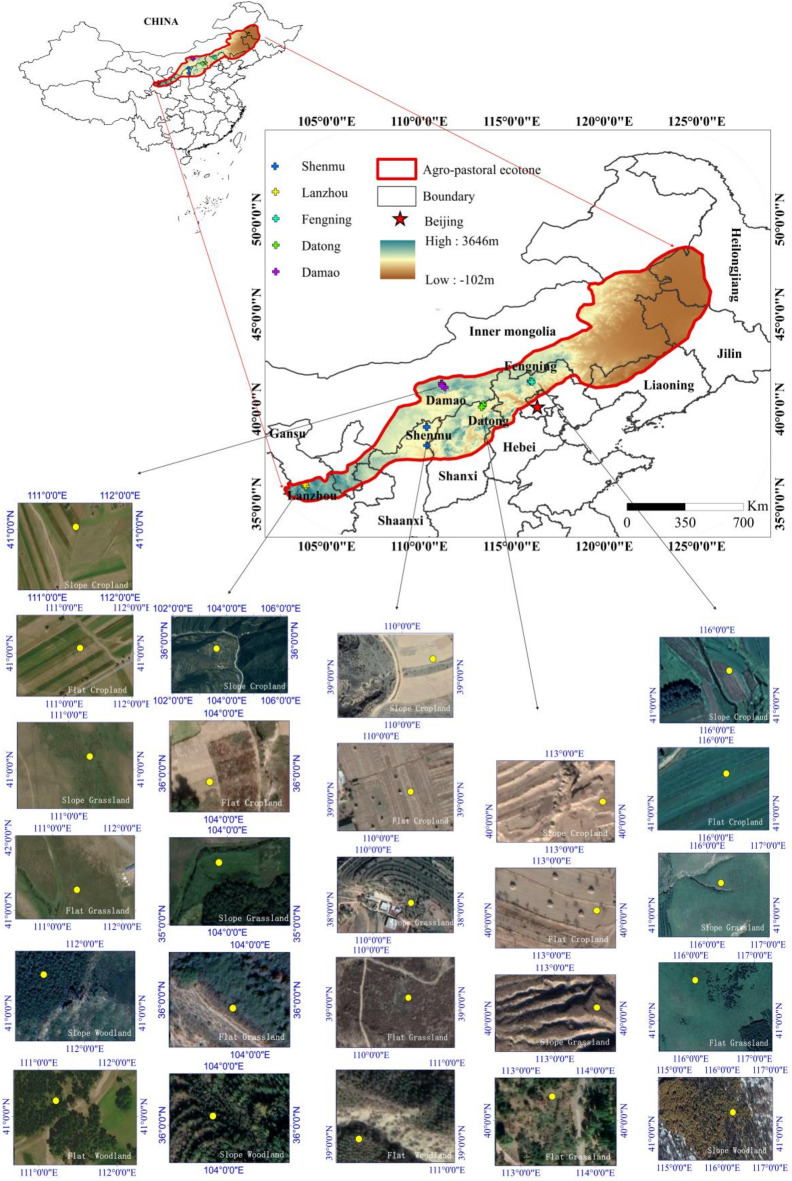
Schematic diagram of sampling points.

The agricultural pastoral transitional zone in northern China spans an area of 726,000 km², accounting for 8.11% of China’s total land area. Situated between 100° to 125° E longitude and 34° to 49° N latitude, this region experiences a transition from a semi-humid continental monsoon climate to a typical arid continental climate. The terrain gradually increases from northeast to southwest, with altitudes ranging from less than 200 m at the lowest point to nearly 4500 m at the highest point. The annual average temperature falls between 2 and 8 °C, while the annual average precipitation ranges from 250 mm to 500 mm. Precipitation exhibits significant variation, with approximately 80% occurring during summer and autumn. Conversely, spring and eastern seasons are relatively dry, often subjected to strong winds. The region’s terrain, landforms, and land use patterns are notably complex. Loess hills dominate the southwestern part, primarily utilized for agriculture, while plateau terrain characterizes the central area, accommodating a mix of agriculture and animal husbandry. The northern region is predominantly grassland, with animal husbandry as the primary focus. Climate conditions and human disturbances contribute to soil and water erosion in this region, typifying it as a wind-water composite erosion belt area. For this study, five regions—Fengning, Datong, Damao, Shenmu, and Lanzhou were selected as research areas for investigating combined wind and water erosion. Table 1 provides some basic information about these regions, Fig. 1 shows the location of the sampling area.

**Table 1:** Basic information of the sampling area in the study area.

Study area	Location	Soil type	Average altitude (m)	Average annual precipitation (mm)	Annual average wind speed (m s^− 1^)	Number of windy days (days)	Mean annual temperature (℃)
Fengning	N40°35′-42°0′ E115°14′-117°24′	Chestnut soil, meadow soil	2000	470	4.3	55	1
Datong	N39°03′-40°44′ E112°34′-114°33′	Coarse bone soil	1530	400	2.2	38.3	6.8
Damao	N41°12′-41°31′ E111°00′-111°20′	Corn brown soil, chestnut soil	1600	255	3	68	3.4
Shenmu	N38°12′-39°27′ E109°37′-110°56′	Coarse bone soil	1183	432	2.2	14	8.5
Lanzhou	N35°34′-37°07′ E102°36′-104°34′	Chestnut calcium soil, brown calcium soil	1750	316	0.8	-	7.5

### Data collection and measurement

#### Selection of sampling areas and field investigation

This study selected five representative regions in northern China’s agro-pastoral ecotone based on their topography, land use patterns, and climatic characteristics: Lanzhou in Gansu Province, Shenmu in Shaanxi Province, Damaoqi in Inner Mongolia Autonomous Region, Datong in Shanxi Province, and Fengning in Hebei Province. These regions encompass diverse environmental conditions, ranging from arid to temperate monsoon climates, and are characterized by typical agro-pastoral transitional features. Lanzhou and Shenmu are located on the Loess Plateau, where the terrain is dominated by steep mountains and deeply incised gullies. These areas are representative of traditional agro-pastoral landscapes, with a mix of agricultural and pastoral activities. Datong is situated on the edge of the North China Plain and features plains and hilly terrain, with diverse land uses including cropland, grassland, and forest. Damaoqi lies in the Inner Mongolia Plateau, where desert grasslands are prevalent, interspersed with patches of cropland. Its arid to semi-arid climate makes it an ideal location for studying alternating wind and water erosion. Fengning experiences a climate transition from arid to semi-humid conditions, with terrain characterized by plains, plateaus, and gully hills. Land use types in this region include grasslands, forests, and croplands. Collectively, these five regions encompass a variety of geomorphic units and land use patterns, providing a comprehensive foundation for investigating the complex erosion dynamics in the agro-pastoral transitional zone.

The primary focus of the field investigation was to accurately locate wind erosion sampling points, ensuring the scientific validity and representativeness of the selected sites. Sampling points were chosen based on their elevation and slope gradient. High and flat terrain was prioritized for wind erosion sampling, as such landforms facilitate wind-driven soil erosion. Higher elevations enhance wind flow and energy, while gentle slopes reduce the potential for water erosion. During field surveys, surface characteristics were observed to further validate the wind erosion features of the sites, such as reduced fine particles and coarser soil texture. Regions exhibiting prominent water erosion features, including sheet erosion, rill erosion, or gully erosion, were strictly excluded to avoid confounding wind and water erosion processes. Each sampling point was precisely geolocated using high-precision GPS, and additional data such as slope gradient, vegetation cover, and soil moisture were recorded to ensure a multidimensional assessment of site characteristics. This systematic and rigorously controlled approach enabled the identification of sampling points that authentically reflect wind erosion features, thereby establishing a robust basis for subsequent soil sampling and erosion analysis.

#### Soil sample collection

This study collected two types of soil samples: ^136^Cs background samples and ^136^Cs erosion/accumulation samples. The selection of background sampling locations was critical, as it directly influenced the accuracy and reliability of the data. Following the sampling standards proposed by Walling^88^, background samples were collected from undisturbed forested and grassland sites within each study area. Prior to sampling, potential locations were screened using remote sensing imagery and historical land-use data to ensure that they had remained unaffected by significant human activities for at least the past 50 years. Furthermore, field surveys were conducted to assess soil profile characteristics, providing additional verification of site stability and representativeness.

The sampling timeframe was determined based on the guidelines outlined in Zapata^89^. For grassland and forest sites, which have stable soil structures, sampling can be conducted year-round. In contrast, cropland soils, which are prone to disturbances from plowing, rainfall, and biological activity, should be sampled during the growing season. To ensure consistency, all samples in this study were collected in July, avoiding periods of heavy rainfall to minimize potential soil displacement. The sampling campaign lasted for one month.

Erosion samples included wind erosion samples and wind-water composite erosion samples, collected from two distinct geomorphic units: high and flat terrain, and slopes. Wind erosion samples were primarily collected from high and flat terrain, as these areas are conducive to wind-driven soil erosion while minimizing the influence of water erosion. Before sampling, wind erosion features, such as reduced fine particles and coarser soil textures, were confirmed through field observations. Wind-water composite erosion samples were collected from slopes adjacent to the wind erosion sampling points to ensure consistent wind conditions. These slope sites represent areas where both wind and water erosion occur, reflecting the dynamic nature of composite erosion processes.

Each study area included two bulk samples and two stratified samples as background samples. Stratified samples were collected in 5 cm increments from the surface to a depth of 30 cm, resulting in six layers per sample. To ensure precision, stratified sampling was conducted using a Dutch hand auger, and the equipment was thoroughly cleaned between samples to prevent cross-contamination. A total of 20 background samples were collected across the five study areas.

^136^Cs erosion/accumulation samples were collected near the background sampling points and covered three land-use types: cropland, grassland, and forest (with adjustments made in regions lacking forest coverage). High and flat terrain samples were collected using an equilateral triangle method, with side lengths of 30–50 m, and one sample was collected at each vertex to ensure spatial representativeness. On slopes, samples were collected from the top, middle, and bottom, with a uniform sampling depth of 30 cm. Slope gradients were measured using a high-precision digital inclinometer to improve data accuracy. In total, 75 erosion/accumulation samples were collected across the five regions (Table 2). For all sampling points, geographic coordinates, slope gradients, land-use types, and vegetation coverage were meticulously recorded during field surveys.

**Table 2:** Soil ^136^Cs content and soil erosion/accumulation modulus in the study area.

Study area	Land use	^137^Cscontent (Bq m^− 2^)	Erosion rate (t km^− 2^a^− 1^)	Slope (°)	Longitude (°)	Latitude (°)
Feng ning	Sloping cropland	1740	1029	6	116.05	41.45
	Flat cropland	2412	59		116.03	41.47
	Slope grassland	5950	-1274	2.5	116.02	41.49
	Flat grassland	2250	114		116.04	41.49
	Slope woodland	3373	-291	30	116.09	41.43
Da tong	Sloping cropland	963	1954	8	113.45	40.19
	Flat cropland	1704	216		113.45	40.18
	Slope grassland	1983	-72	20	113.45	40.24
	Flat grassland	2198	-177		113.37	40.10
Da mao	Sloping cropland	1251	1353	7	111.25	41.23
	Flat cropland	1876	138		111.24	41.23
	Slope grassland	841	489	3	111.21	41.35
	Flat grassland	618	598		111.22	41.33
	Slope woodland	1228	311	6.5	111.39	41.14
	Flat woodland	717	548		111.24	41.23
Shen mu	Slope cropland	216	6282	14	110.40	39.07
	Flat cropland	1400	280		110.41	39.04
	Slope grassland	499	662	18	110.41	38.08
	Flat grassland	675	579		110.41	39.06
	Flat woodland	214	809		110.41	39.05
Lan zhou	Slope cropland	210	7126	15	103.87	35.92
	Flat cropland	1565	252		103.86	35.95
	Slope grassland	151	828	8	103.87	35.93
	Flat grassland	2252	-290		103.87	35.93
	Slope woodland	421	663	40	103.82	36.04

#### Soil sample processing and Cs activity measurement

In this study, soil samples were air-dried under controlled natural conditions to prevent contamination or alterations to their properties. The dried samples were ground using a milling device and sieved through a 2 mm mesh. The coarse fraction (> 2 mm) and fine fraction (< 2 mm) were weighed separately with a precision balance (± 0.01 g accuracy). The activity of ^136^Cs was measured using an ORTEC GMX50P4N high-purity germanium γ detector coupled with a DSPEC-ji-2.0 digital multichannel analyzer. Before sample measurement, the detection system was calibrated using certified reference materials, and regular recalibration was performed to ensure consistent measurement accuracy. The system error was maintained within ± 5% at a 95% confidence level.

To ensure reliable γ-ray statistical counts, each sample was measured for no less than 10 h, guaranteeing the precision of the results. The relative comparison method was employed to calculate ^136^Cs activity. This involved determining the net area of the full-energy γ-ray peak and combining it with the emission probability and detector efficiency to calculate the activity of the nuclide. The activity value was then normalized to the sample mass to determine the specific activity (Bq·kg⁻¹). Each sample was analyzed three times, and the average value was used as the final result to minimize random errors. Finally, the activity value obtained was divided by the sample mass to calculate the specific activity of the nuclide (Bq·kg^− 1^). The calculation formula is as follows:

1 $$\:A={C}_{e}\frac{{A}_{S}{F}_{1}}{{F}_{2}T{m\:e}^{-\lambda\:\varDelta\:t}}$$

2 $$\:{C}_{e}=A/{a}_{s}.$$

3 $$\:{F}_{1}=\frac{\lambda\:{T}_{c}}{1-{e}^{-\lambda\:{T}_{c}}}\:\:\:$$

In the formula:*A* is Specific activity of the nuclide (Bq·kg^− 1^). ${C_e}$is scale factor, $A'$is the scale source nuclide activity (Bq), ${a_s}$is Net area count rate (s^− 1^) of one or more weighted characteristic allochthonous peaks of the selection, ${A_s}$is Net area (counts) of characteristic nuclide peaks obtained from the beginning to the end of the measurement sample, ${F_2}$is the sample self-absorption correction factor with respect to the scale source γ,${F_2}$=1; *T* is the sample measurement live time (s),* m* is Measurement of the mass of the sample (kg), $\Delta t$is Nuclide decay time, i.e. the time interval (s) between the moment of sampling and the moment of sample measurement, $\lambda$is the radionuclide decay constant (s^− 1^), ${F_1}$is Decay correction factor during sample measurement if the half-life of the analyzed nuclide is greater than 100 compared to the time of the sample measurement, ${F_1}$ = 1; ${T_c}$is the true time of measurement of the sample (s).

#### Model application

The cropland erosion rate was estimated using the Mass Balance Model II (MBMII) proposed by Walling and He^90^. This model, based on the principle of mass balance, is suitable for estimating multi-year average soil erosion rates since the 1960s. The MBMII model not only quantifies soil loss but also accounts for the redistribution of soil within the landscape, making it ideal for erosion studies in complex terrain. With low data requirements and high adaptability, this model has been widely used by researchers to estimate cropland erosion rates^91^^–^^93^. Therefore, this study applied the MBMII model to estimate cropland erosion rates. The differential formula for the model is as follows:

4 $$\:\frac{dA\left(t\right)}{dt}=(1-\varGamma\:)I\left(t\right)-(\lambda\:+P\frac{R}{{d}_{m}})A\left(t\right)$$

In the formula:

5 $$\:\varGamma\:=P\gamma\:(1-{e}^{-R/H})$$

$\:\frac{dA\left(t\right)\:}{dt}$ is the rate of change of ^136^Cs content in soil per unit time.

$\:A\left(t\right)$Meaning the amount of cesium remaining in the soil from the year of deposition (1954) until the year of sampling, year by year the amount of ^136^Cs deposited in the soil, year by year the total amount of ^136^Cs remaining in the soil is equivalent to the amount of ^136^Cs remaining in the erosion/accumulation samples measured in the sampling year, Bq m^− 2^;

$\:I\left(t\right)$ is the ^136^Cs deposition of cesium from the year of deposition (1954) until the sampling year, year by year, Bq/m^2^; the sum of the ^136^Cs deposition of each year is equal to the ^136^Cs background value of the sampling year, Bq m^− 2^.

$\:\varGamma\:$ is Loss rate of newly deposited ^136^Cs before mixing into the till layer. It has also been demonstrated that 98% of the Cs deposited to the surface will be absorbed by the soil or soil organic matter^94^ and rapidly in two or three minutes instantaneously^95^, the rainfall interval in the present study area is generally long, and the amount of secondary rainfall is relatively small, so that the loss rate of ^136^Cs is negligible, and the value taken in this study is 0.

$\:P$ is the particle correction factor, the cultivated land has been tilled for many years, the soil particles have been fully mixed, so its loss and deposition process can be regarded as homogeneous, and the particle sorting effect is not significant; therefore, the value of 1 is taken in this study。

$\:{d}_{m}$ is the cumulative mass depth of soil in the tillage layer( kg·m^− 2)^, and the specific calculation formula is = tillage depth × soil bulk weight, after investigation, the tillage depth in this study area is mostly 20 cm, and the bulk weight ranges from 1.2 to 1.35 g·cm^− 3^.To ensure the accuracy of input parameters, bulk density was measured multiple times at each sampling location, and outliers were identified and removed using statistical methods. Consistency of measurements was verified through comparisons with historical data from similar regions. The tillage depth was also cross-checked against local farming records and field observations to minimize potential biases in parameter estimation.

$\:R\:$is the annual erosion rate, kg·m^− 2^·a^− 1^; $\:\lambda\:$=0.9773, ^136^Cs decay constant.

The erosion rate for forested and grassland areas was calculated using the Profile Distribution Model^96^. This model, based on erosion and deposition characteristics at different depths within soil profiles, utilizes radiogenic nuclides (such as ^136^Cs) and geochemical data to accurately quantify long-term erosion processes. It is well-suited for complex terrain, effectively reflects natural erosion trends, and is capable of assessing erosion over extended time scales. Additionally, the model is simple in form, requires few parameters, and is easy to calculate^97^. Due to these advantages, it has been widely adopted by numerous researchers^65,98^^–^^101^.

The model is as follows:

6 $$\:X={X}_{0}\cdot\:{e}^{-\lambda\:\cdot\:h\cdot\:(T-1963)}$$

where *X* is the current Cs area activity (Bq·m^− 2^) at the sampling site; *X*_0_ is Cs background value (Bq·m^− 2^); *λ* is Cs background value profile distribution morphology parameter (obtained from Cs content distribution in the profile); *h* (cm·a^− 1^) is the annual average soil wind erosion thickness since 1963; and *T* is the sampling year.

### Distinction between wind and water erosion rates

In this study, soil samples were collected from two typical geomorphic environments—high flatlands and slopes—to distinguish the rate ratios of wind and water erosion using scientific methods. On high flatlands (with a slope of less than 2 degrees), the elevated terrain and minimal water accumulation capacity theoretically allow hydraulic erosion to be neglected, making these sites suitable for measuring wind erosion rates. At slope sampling points, both wind and water erosion may occur, and the measured erosion rate represents the composite erosion rate of wind and water. Under this framework, we assumed spatial uniformity in wind erosion rates across the entire area to ensure the comparability of wind erosion rates. Therefore, a differential method was employed to distinguish wind and water erosion rates: the composite erosion rate observed at the slope sampling point was subtracted by the wind erosion rate recorded at the adjacent high flatland sampling point, with the resulting difference representing the water erosion rate.

Furthermore, the wind and water erosion rates obtained in this study were cross-validated with field measurement data from neighboring regions. The erosion rates estimated using ^136^Cs in this study were compared with observational data from sand collectors for wind erosion and runoff plot data for water erosion from related research. Differences in measured values and correlation analyses were performed to confirm the reliability of the results.

### Statistical analysis of data

In this study, SPSS software was used to perform statistical analysis on erosion rate data across different land use types to uncover the erosion differences and underlying driving factors for each land type. First, using the descriptive statistics module, we calculated the mean, range, standard error, and 95% confidence interval for sample data from cropland, grassland, and woodland to provide a comprehensive description of erosion intensity characteristics for each land type. The mean values were then used to evaluate the differences in erosion intensity across various land use types. Additionally, the SPSS frequency analysis module was employed to identify the distribution patterns and frequency characteristics of water erosion modulus in areas with similar slopes within and around the study region, further validating the reliability of the water erosion results in this study.

## Results

### Determination of ^136^Cs background value

**Fig. 2:**
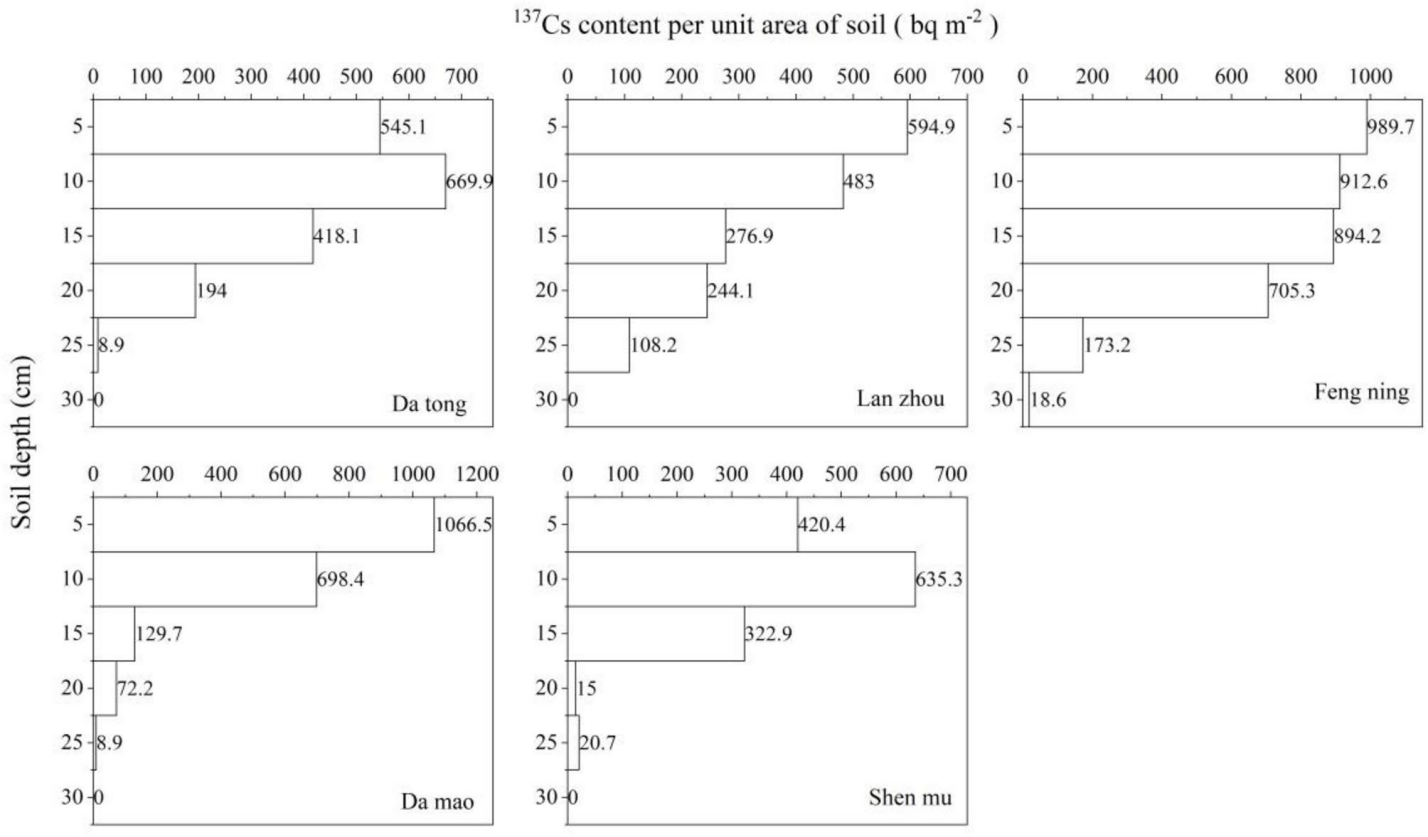
Characterization of ^136^Cs activity profiles of background samples.

In this study, the background samples were initially chosen to be sampled in the forest and grassland without human disturbance according to the background value sample sampling technique standard of Walling D.E^88^, aiming to ensure conditions of no erosion and no accumulation. Subsequently, the ^136^Cs background values were simulated according to the calculation model of global ^136^Cs background values established by Walling D.E and He^90^, and a comparison of the simulated and measured values is presented in Table 3. The measured and simulated background values of Datong and Damao are very close to each other. However, the measured value of Fengning exceeds the simulated value by 1.3 times. This discrepancy may be attributed to the fact that the model evaluates the ^136^Cs deposition value based on precipitation roughly calculated from latitude and longitude information, thus ignoring the difference in precipitation caused by altitude elevation. The sampling point of Fengning’s background value is approximately 2100 m above sea level, significantly higher than the 1000–1500 m in Datong and 1000 m in Da mao Banner, suggesting that the difference in precipitation due to altitude could be the primary reason why the simulated value of Fengning is lower than the measured value. Moreover, the measured value of Shenmu County is 1.35 times that of the simulated value. Notably, the measured value of 1480 Bq·m^− 2^ corrected to 2000 in Shenmu County closely matches the measured value of 1580 Bq·m^− 2^ reported by Li^102^, and the measured value of Lanzhou exceeds the simulated value by 1.78 times. Furthermore, regarding the distribution characteristics of ^136^Cs profiles(Fig. 2), the changes of ^136^Cs profiles with depth in Feng ning, Damao, and Lanzhou belong to the exponentially decreasing form, which is commonly adopted by most scholars^103^^–^^106^. Conversely, the distribution of Datong and Shenmu profiles is characterized by a sharp peak shape, a pattern also recognized as relatively frequent^91,92^. Thus, judging from the comparison between measured and simulated values and the distribution pattern, the background value of ^136^Cs determined in this study is deemed reasonable.

**Table 3:** Comparison of measured values of ^136^Cs background and walling model simulation (Bq m^− 2^).

Study area	Measured value	Model simulation	Measured value/simulated value
Fengning	2462	1820	1.35
Datong	1836	1868	0.98
Damao	1967	1621	1.21
Shenmu	1414	1085	1.30
Lanzhou	1707	958	1.78

The accuracy of background values is crucial. In this study, we first applied the global ^136^Cs background value calculation model developed by Walling and He^90^, referred to here as the WHM model, to simulate background values in the study area, and then compared the simulated values with the measured values (Table 3). The comparison showed that the measured background values in Datong and Damao Banner closely matched the model-simulated values, indicating the model’s high applicability in these regions. However, the measured values in Fengning were 1.3 times higher than the simulated values, likely due to the model’s reliance on latitude and longitude for estimating precipitation, without accounting for altitude effects. The elevation at the Fengning sampling site is approximately 2100 m, higher than that in Datong (1000–1500 m) and Damao Banner (1000 m), which likely increases precipitation and, consequently, the ^136^Cs deposition rate. This difference in precipitation due to altitude is likely the primary reason for the higher measured values in Fengning compared to the model.

Additionally, the measured values in Shenmu were 1.35 times higher than the simulated values. Adjusting Shenmu’s data to the year 2000, we obtained a measured value of 1480 Bq·m^− 2^, consistent with the 1580 Bq·m^− 2^ reported by Li^102^, further validating the reasonableness of background values in the Shenmu area. In Lanzhou, Gansu Province, the measured ^136^Cs value was 1.78 times the WHM model’s simulated value, aligning with results from other areas on the Loess Plateau. For example, the measured values in Tianshui and Xifeng, Gansu Province, were 1.82 and 1.56 times higher than the simulated values^101^, respectively. These findings support Wang^53^ observation that the WHM model often underestimates values in certain areas, primarily because it does not fully account for regional specificity or all factors influencing precipitation, suggesting that the model’s accuracy needs improvement^53,107^.

In terms of profile distribution, the ^136^Cs profiles in Fengning, Damao, and Lanzhou displayed a typical exponential decay pattern with increasing depth, a distribution widely recognized globally^103^^–^^106^. Conversely, the profiles in Datong and Shenmu showed a peak pattern (Fig. 2), often associated with short-term heavy rainfall or natural soil deposition processes, a form frequently mentioned in the literature^108,109^. Thus, based on profile distribution patterns, the ^136^Cs background profile distribution in this study aligns with Du regression findings, derived from extensive data on non-cultivated soil profiles^110^, further verifying the scientific validity of the background values determined in this study.

In summary, through comparison of measured and simulated values, along with validation of profile distribution patterns, this study ensures the scientific robustness of ^136^Cs background values, providing a reliable data foundation for accurately calculating wind and water erosion rates.

### Soil erosion rates under different land uses

**Fig. 3:**
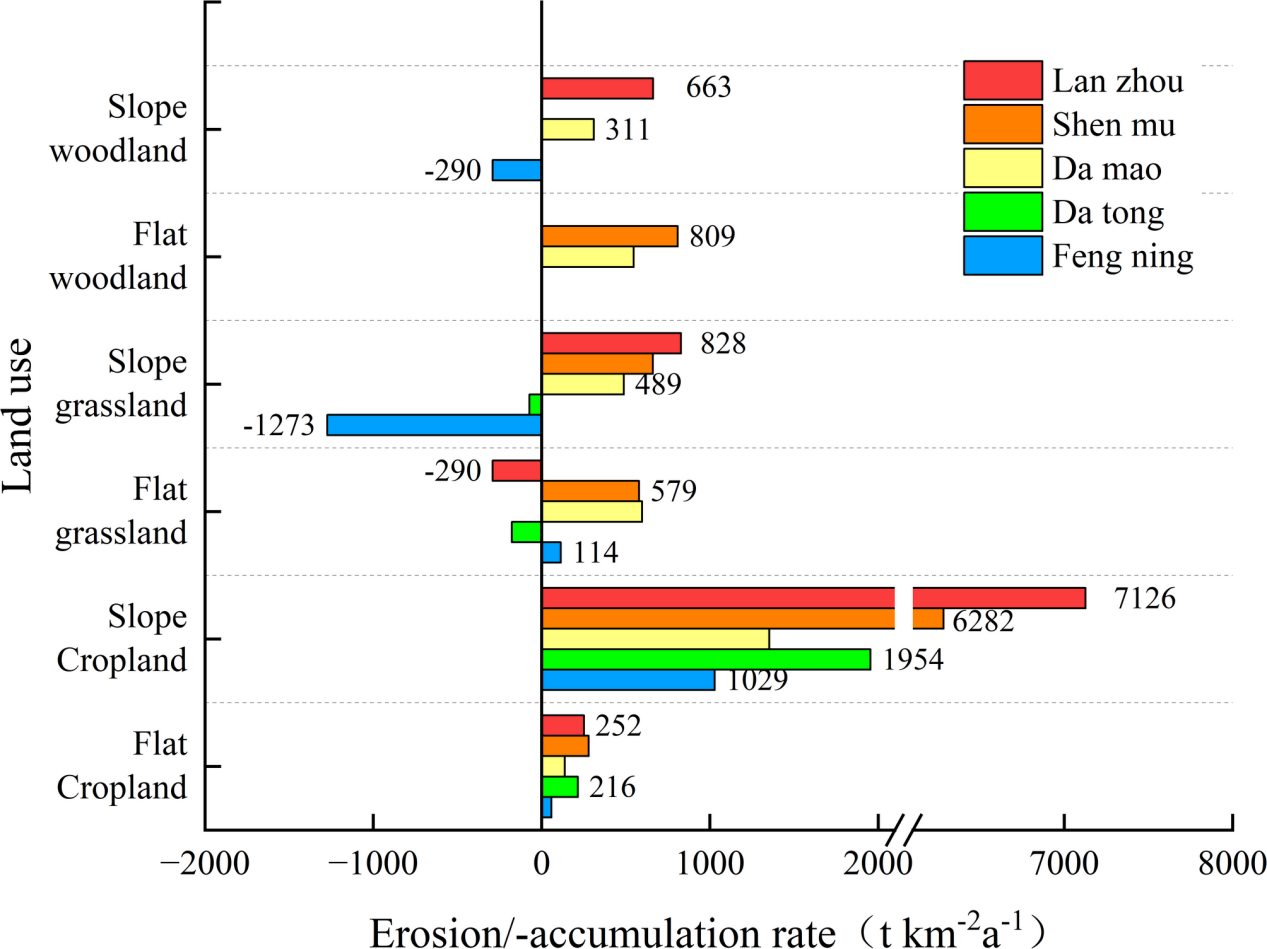
Erosion rate of different land use.

**Fig. 4:**
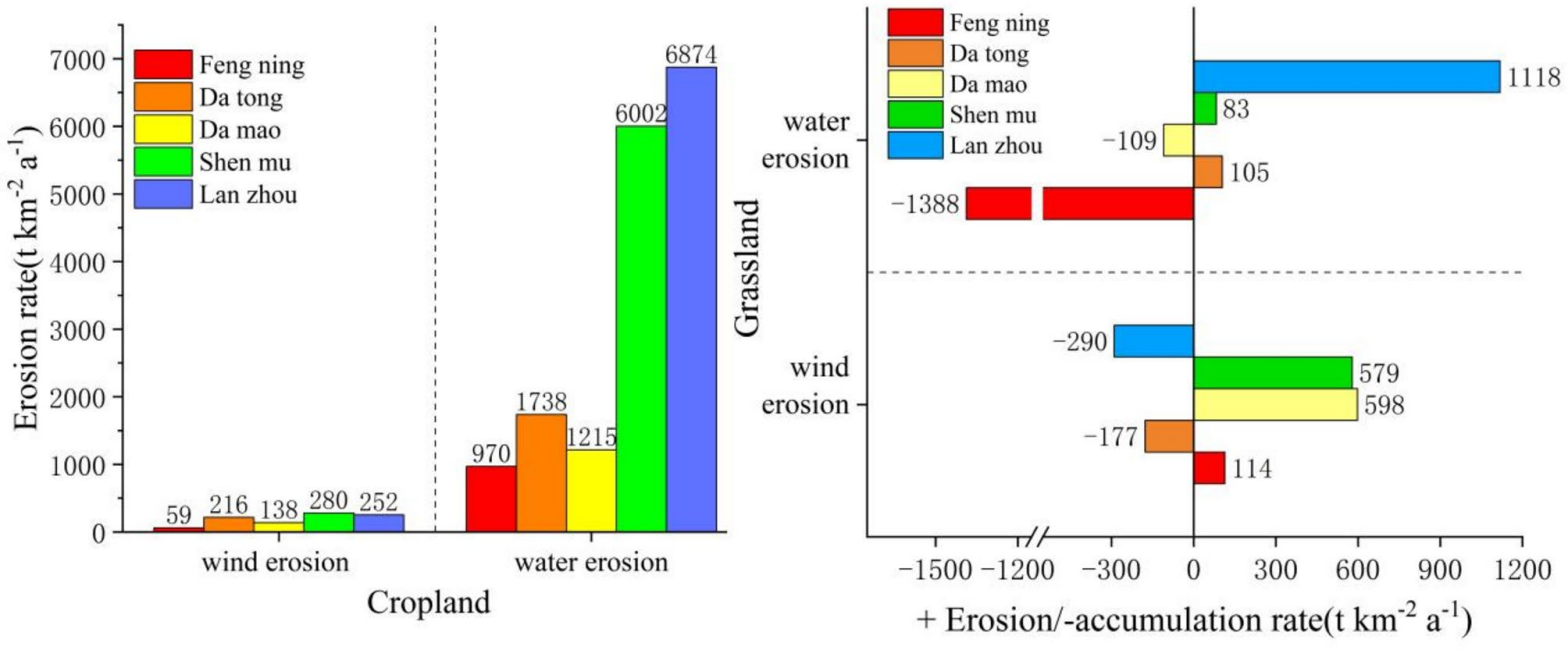
Rate of wind and water erosion in agro-pastoral ecotone.

According to the calculation results (Table 2; Fig. 3), the erosion rate of cropland in the agricultural and pastoral areas ranged from 59 to 7126 t·km^− 2^·a^− 1^, with a 95% confidence interval of 478–3696 t·km^− 2^·a^− 1^, and an average erosion rate of 1868.9 t·km^− 2^·a^− 1^. For grassland, the erosion rate ranged from − 290 to 828 t·km^− 2^·a^− 1^, with a 95% confidence interval of 54.7–622.0 t·km^− 2^·a^− 1^, and an average erosion rate of 145 t·km^− 2^·a^− 1^ (Fig. 4). Except for the grassland of Feng ning slope, the average erosion rate of grassland in other regions was 303 t·km^− 2^·a^− 1^. The 95% confidence interval of the erosion rate of forest land was 49–698 t·km^− 2^·a^− 1^, with an average erosion rate of 408 t·km^− 2^·a^− 1^. Overall, significant differences were observed in the erosion rates of different land use modes. Cropland exhibited higher erosion rates compared to woodland and grassland. The average erosion rate of cropland was approximately 6 times that of grassland and about 4.5 times that of woodland. Specifically, the average erosion rate of cropland in the north-central regions such as Damao, Datong, and Feng ning was 791 t·km^− 2^·a^− 1^, while in the southwestern hilly regions such as Lanzhou and Shenmu, it was relatively high, reaching 3485 t·km^− 2^·a^− 1^. The noticeable difference in erosion rates between regions can be attributed to the fact that Lanzhou and Shenmu belong to the loess hills and gullies area, characterized by extensive slopes. The cropland sample sites in these regions have slopes ranging from 10–15°, making them more susceptible to water erosion during the precipitation season, resulting in a relatively high erosion rate of the sloping cropland in these areas, with a mean value of 6704 t·km^− 2^·a^− 1^. In contrast, the cropland sampled in the other three areas had slopes ranging from 6–8°, with erosion rates of less than 2000 t·km^− 2^·a^− 1^. It is evident that the slope of the study area plays a crucial role in cropland erosion, highlighting the significance of precipitation and topographic conditions in influencing cropland erosion in this region. In terms of erosion intensity, according to the standards of the Ministry of Water Resources of China, the soil erosion degree of cultivated land in the study area is classified as moderate to severe, while grassland and forest land are categorized as mild erosion states.

Based on the calculation results (Table 2; Fig. 3), erosion rates across different land use types in the agricultural-pastoral ecotone show significant variation. Erosion rates for cropland range from 59 to 7126 t·km^− 2^·a^− 1^, with a 95% confidence interval of 478–3696 t·km^− 2^·a^− 1^ and an average rate of 1868.9 t·km^− 2^·a^− 1^. In contrast, grassland erosion rates are lower, ranging from − 290 to 828 t·km^− 2^·a^− 1^, with a 95% confidence interval of 54.7–622.0 t·km^− 2^·a^− 1^ and an average rate of 145 t·km^− 2^·a^− 1^ (Fig. 4). It is worth noting that, excluding sloped grasslands in Fengning, the average erosion rate for grasslands in other regions is 303 t·km^− 2^·a^− 1^. Forestland erosion rates vary between 49 and 698 t·km^− 2^·a^− 1^, with a 95% confidence interval of 49–698 t·km^− 2^·a^− 1^ and an average of 408 t·km^− 2^·a^− 1^.

Overall, erosion rates are significantly higher in cropland compared to grassland and forestland. The average erosion rate in cropland is approximately six times higher than that in grassland and 4.5 times higher than that in forestland. Specifically, cropland erosion rates vary considerably across regions: in northern and central regions such as Damao Banner, Datong, and Fengning, the average cropland erosion rate is 791 t·km^− 2^·a^− 1^; however, in southwestern hilly areas such as Lanzhou and Shenmu, cropland erosion rates are notably higher, averaging 3485 t·km^− 2^·a^− 1^ approximately 5 times the rate in the northern and central areas. This difference is largely attributable to the loess hills and gully terrain in Lanzhou and Shenmu, where slopes are prevalent, with cropland sites having slopes of 10–15°. During the rainy season, these sloped croplands are highly susceptible to water erosion, leading to significantly elevated erosion rates, with an average of 6704 t·km^− 2^·a^− 1^. In contrast, cropland sites in the northern and central regions are situated on gentler slopes (6–8°) with erosion rates consistently below 2000 t·km^− 2^·a^− 1^.

These results indicate that slope and precipitation play critical roles in cropland erosion processes. Areas with steeper slopes are more prone to water erosion during concentrated rainfall periods, thus significantly increasing erosion intensity. Consequently, topography and precipitation patterns are key factors in determining cropland erosion levels in this region.

From the perspective of erosion intensity, according to the soil erosion standards set by the Ministry of Water Resources of China, cropland erosion in the study area generally falls within moderate to severe categories, whereas erosion in grassland and forestland is relatively mild, remaining in the slight erosion category. These findings underscore the importance of rational land use and terrain management in mitigating soil erosion.

### Proportion of wind-water composite erosion rates

In previous studies on wind-water composite erosion, it has been commonly assumed that only wind erosion occurs on relatively high and flat terrain, while hydraulic erosion is negligible. However, on sloping surfaces, both wind and hydraulic erosion can occur simultaneously^111^. In this study, we made a preliminary assessment of the proportion of these two erosion processes, wind and hydraulic, based on this assumption.We selected flat croplands in the agricultural and pastoral intertwined area as our study area. The distribution of wind erosion rates ranged from 59 to 280 t·km^− 2^·a^− 1^, with a mean value of 189 ± 90.11 t·km^− 2^·a^− 1^. In comparison, erosion intensity was significantly greater in sloping croplands due to the occurrence of both wind and water erosion. The erosion rate ranged from 970 to 1738 t·km^− 2^·a^− 1^ on sloping croplands with slopes of 6–8° and approximately 6000–6874 t km^− 2^a^− 1^ on those with slopes of 10–15°. Based on our assumed conditions, we subtracted the total erosion rate from the wind erosion rate on flat croplands in each area to obtain the hydraulic erosion data. According to our results (Table 4), the distribution of hydraulic erosion rates on sloping croplands with different slopes ranged from 970 to 6874 t·km^− 2^·a^− 1^. In gently sloping croplands of 6–8°, the ratio of wind to hydraulic erosion rates was 1:8 − 1:16, while in sloping croplands of 10–15°, the ratio was 1:21 − 1:27. This indicates that the wind erosion rate is significantly lower than the water erosion rate in the agro-pastoral ecotone of northern China. For woodland and grassland, we observed both erosion and accretion at each sample site. The maximum erosion rate was 828 t·km^− 2^·a^− 1^, while the maximum accumulation rate was 1274 t·km^− 2^·a^− 1^. Compared to cropland, the erosion intensity of woodland and grassland was lower, and the difference in erosion due to topographic slope was not significant. Therefore, distinguishing the contributions of wind and hydraulic erosion rates in woodland and grassland proved relatively challenging in this study and requires further investigation.

**Table 4:** Comparison of wind and hydraulic erosion rates in the study area (t km^− 2^a^− 1^).

	Fengning		Datong		Damao		Shenmu		Lanzhou	
	C	G	C	G	C	G	C	G	C	G
Total	1029	-1274	1954	-72	1353	489	6282	662	7126	828
Wind erosion	59	114	216	-177	138	598	280	579	252	-290
Water erosion	970	-1388	1738	105	1215	-109	6002	83	6874	1118
Wind erosion/water erosion	1:16	1:-12	1:08	-1:0.6	1:09	1:-0.2	1:21	01:00.1	1:27	-1:4

‘-’ is accumulation, C is cropland; G is Grassland.

## Discussion

### Range of soil erosion modulus in agricultural and pastoral intertwined areas

In this study, we conducted a detailed analysis of erosion modulus in agro-pastoral ecotone, highlighting the impact of slope gradient on soil and water loss rates. Data analysis revealed a significant increase in soil and water loss rates with steeper slope gradients, aligning with previous research and emphasizing the relationship between soil erosion processes and topographic slope.

Gentle slopes (6–8°) exhibited lower soil and water loss rates, typically below 2000 t·km⁻²·a⁻¹. This phenomenon is attributed to reduced runoff velocity and weaker erosion intensity on gentler slopes. These findings are consistent with Li’s study^111^ in Weinan County, Shaanxi Province, demonstrating that farmlands on gentle slopes are primarily controlled by runoff processes, resulting in relatively low soil and water loss rates.

However, when the slope increased to 10–15°, the soil and water loss rate rose significantly^112^, particularly in the Loess Hilly Region, where the loss rate reached 6000–6874 t·km⁻²·a⁻¹. This observation highlights a positive relationship between slope gradient and the erosive potential of water flow. In steeper areas, water flow velocity is higher, and soil particles are more easily transported by the flow, leading to a substantial increase in soil and water loss rates. These findings align with those of Liu, who documented soil and water loss rates of 6396–7603 t·km⁻²·a⁻¹ in farmlands with slopes of 8–15°, further validating the significant impact of slope gradient on soil erosion^112,113^.

Additionally, this study observed that in areas with slopes > 15°, the rate of soil and water loss further increased. In the sloping lands of the Loess Plateau, soil and water loss rates specifically reached 1599-3148.2 t·km⁻²·a⁻¹^114^. This trend is consistent with previous research, indicating that in areas with steep slopes, the combined effects of wind and water exacerbate soil loss due to increased surface roughness and reduced vegetation cover. These findings not only corroborate earlier studies but also provide more reliable data to explore the impact of slope gradient on soil erosion processes.

**Fig. 5:**
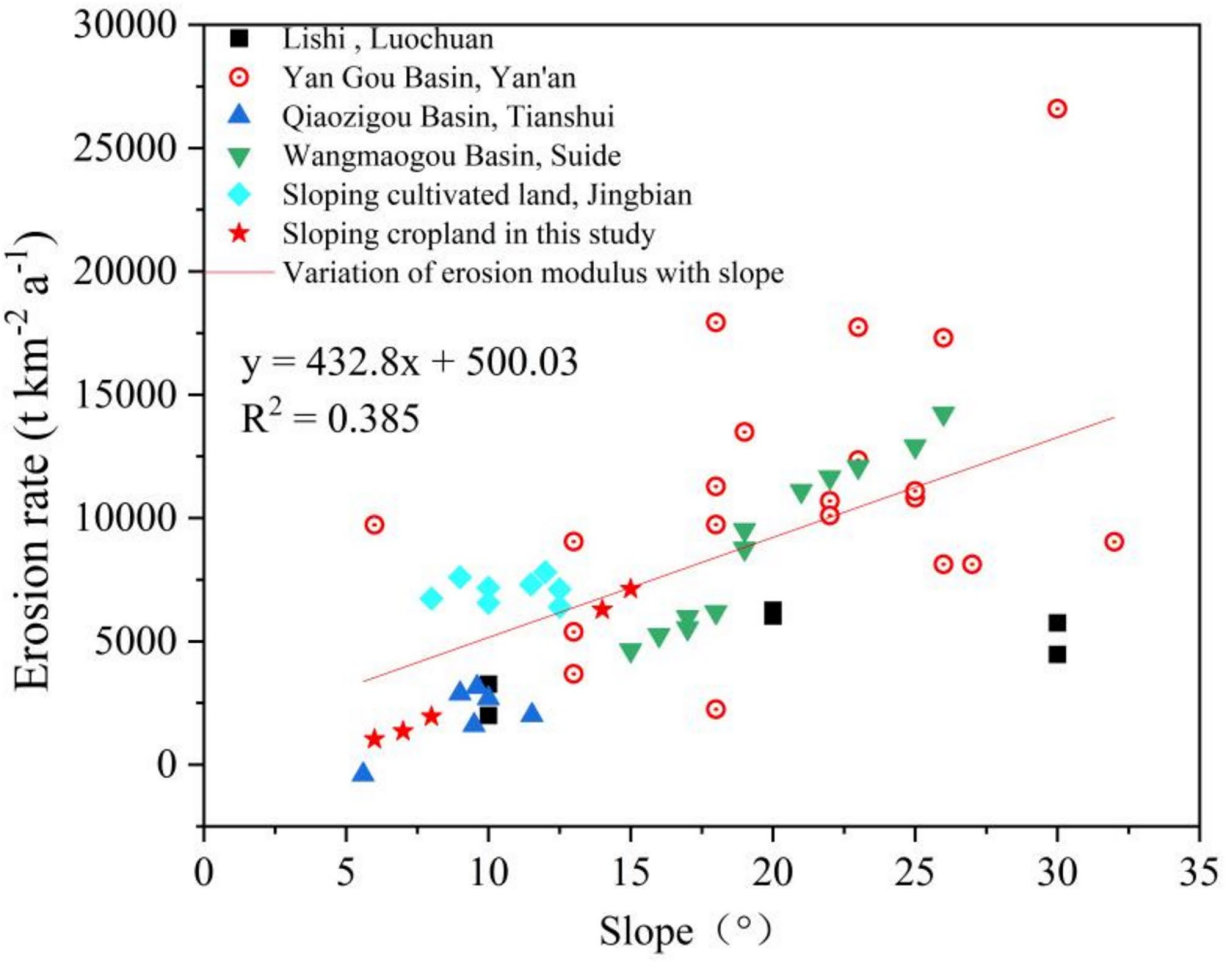
Soil erosion rates in the vicinity of the study area based on the ^136^Cs method.

By comparing results from regions with varying slopes, we also identified a strong linear relationship between slope gradient and soil and water loss modulus. As shown in (Fig. 5),Specifically, in areas with larger slopes (> 15°), the soil and water loss rate increased significantly, consistent with the variation pattern of ¹³⁷Cs content. As the slope increased, the loss of ¹³⁷Cs also rose markedly, indicating that steeper slopes result in greater loss of surface soil radioactive elements such as ¹³⁷Cs. As an important indicator of soil and water loss, the variation in ¹³⁷Cs content effectively reflects the intensity of soil erosion. Therefore, the strong correlation between slope gradient and ¹³⁷Cs content further verifies the reliability of the ¹³⁷Cs method for measuring soil and water loss modulus^115,116^.

These findings demonstrate that the influence of slope gradient on soil and water loss is not a simple linear increment^117^ but rather the result of multiple factors acting in concert, including topographic complexity, the erosive power of water flow, and changes in vegetation cover^118^. Therefore, future soil and water conservation efforts should consider the integrated effects of slope gradient, rainfall intensity, and vegetation restoration^119,120^ to develop more precise conservation measures that mitigate the adverse impacts of soil erosion on agricultural production and ecological environments.

## Validation of water erosion rates

Field observation data serve as a critical tool for validating the scientific validity and reliability of indirect observation methods. They also form the essential foundation for calibrating model parameters and improving the accuracy of erosion rate estimation. Among these methods, runoff plots are widely recognized as one of the most classic approaches in water erosion research due to their quantitative precision and localized focus^59^. Since Ewald Wollny first introduced runoff plots in slope studies in the late 19th century, this method has become a core tool for investigating the relationships among rainfall, runoff, and soil erosion. The United States began applying runoff plots in 1912, and China adopted this method in 1922 to delve into the mechanisms linking rainfall, slope gradient, and soil erosion^121^.

**Fig. 6:**
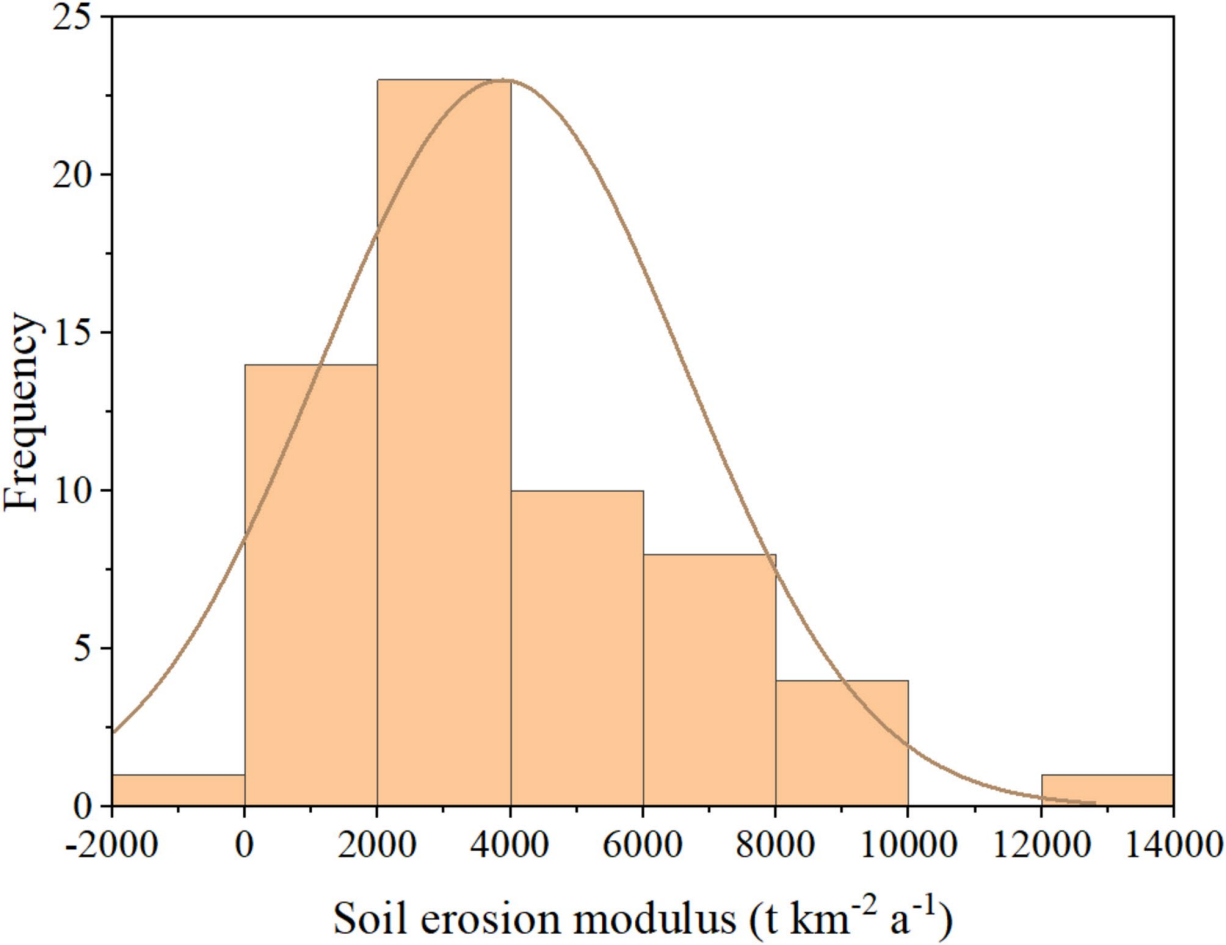
Frequency statistics of soil water erosion rate in the study area.

Based on long-term observations from runoff plots in the study area, comprehensive water erosion rate data were obtained. Results indicate that the erosion modulus ranges from 10 to 12,890 t·km⁻²·a⁻¹, reflecting significant spatial heterogeneity in erosion intensity across the region. Further analysis revealed that, in plots with slope gradients concentrated between 5° and 18°, 95% of the erosion modulus values ranged from 2605 to 4145 t·km⁻²·a⁻¹, with an average of 3284 t·km⁻²·a⁻¹ (Fig. 6). These findings not only delineate the primary distribution range of regional water erosion rates but also provide scientific insights into the relationships among slope gradient, rainfall conditions, and water erosion.

The differences in erosion modulus are primarily attributed to the combined effects of soil type, land use practices, and slope gradient. Soil type is one of the key influencing factors. For example, Jin et al. found that, under identical slope conditions, erosion modulus in loess plots was 4–7 times higher than that in sandy soil plots^122^. Land use practices further amplified the variation in erosion modulus. Guo et al. reported that the erosion modulus of bare land plots was significantly higher than that of cultivated land plots, with bare land erosion modulus reaching 5039 and 12,412 t·km⁻²·a⁻¹ in 2012 and 2013, respectively, compared to 4198 and 8945 t·km⁻²·a⁻¹ for cultivated land plots^122,123^. These findings underscore the critical role of land use practices in modulating erosion rates under similar rainfall intensities.

The influence of slope gradient on water erosion rates exhibits nonlinear characteristics, which vary significantly with regional and environmental conditions. Wang reported that gully erosion was most pronounced in the North China Plain when slope gradients ranged between 10° and 15°. In contrast^124^, Tang observed peak gully erosion intensities in the black soil region of Northeast China at slope gradients of 15° to 20°^125^. However, Liu and Zhang found that, on the Loess Plateau, gully erosion intensity began to decrease when slope gradients exceeded 18°, and extreme slopes (> 30°) showed further reductions due to flow dispersion effects^126,127^. These findings align with the observed patterns within the study area, where slopes range from 0.5° to 18.66°, confirming that slope gradient effects significantly influence the spatial distribution of erosion modulus. Table 5 presents the relevant data.

To further validate the reliability of the estimated water erosion rates, data from plots with slope gradients of 6° to 15° were sampled for analysis. Results showed an average erosion modulus of 2,715 t·km⁻²·a⁻¹, with a 95% confidence interval ranging from 1990 to 3517 t·km⁻²·a⁻¹. This validation aligns closely with the observed range in this study, reflecting the compounded effects of slope gradient, rainfall intensity, and land use practices on water erosion processes.

Rainfall erosivity plays a critical regulatory role in water erosion modulus variation and exhibits a significant compounding effect with slope gradient. For example, studies by Jin and Guo demonstrated that, in plots with similar slope gradients, the erosion modulus increased from 2500 t·km⁻²·a⁻¹ during 1980–1989, when rainfall intensity was concentrated at 150 mm, to 4071 t·km⁻²·a⁻¹ in 2012–2013, when rainfall intensity increased to 210 mm^123,124^. This clearly indicates that rainfall intensity not only amplifies the influence of slope gradient on erosion but may also alter the spatial patterns of water erosion rates by enhancing runoff erosivity.

In summary, this study validated the scientific robustness of water erosion rates through runoff plot data and elucidated the spatial distribution and dynamic variations of water erosion modulus by integrating the effects of soil type, slope gradient, and rainfall intensity. Future research should aim to explore water erosion processes under complex conditions by integrating long-term monitoring data with dynamic models to provide more precise scientific support for regional land management and soil conservation practices.

**Table 5:** Observations of water erosion by the runoff plot method in the loess plateau region.

Location	Land use/soil type	Year of measurement	Average slope	Modulus of erosion (t km^− 2^a^− 1^)
Suide county	Sesame beans	1954, 1956–1958	18.66	5295.39
Ansai county	Buckwheat	1987–1991	18.66	3097.99
	Grain	1987–1991	18.66	3257.45
	Wheat	1987–1991	18.66	3474.73
	Millet	1987–1991	18.66	3888.26
	Soybean	1987–1991	18.66	4259.74
	Bare ground	1987–1991	18.66	4523.76
	Bare ground	1983–1988	18.66	5490.48
Junggar banner	Sandy soil	1987–1989	6	827.7
	Loess	1987–1989	6	2669.8
	Arsenic sandstone soil	1987–1989	6	3157.6
	Windswept sandy soil	1983–1985	9	421.9
	Loess	1983–1985	9	3019.5
	Arsenic sandstone	1983–1985	9	4279.8
	Loess	1985–1990	17	2670.8
	Arsenic sandstone soil	1985–1990	17	4830.1
	Loess	1985–1990	0.5	10
	Loess	1985–1990	6	1210
	Loess	1985–1990	9	1960
	Loess	1985–1990	12	2360
	Loess	1985–1990	15	3740
	Arsenic sandstone soil	1988–1990	6	2860
	Arsenic sandstone underlain by thin layers of loess	1988–1990	9	^137^0
	Arsenic sandstone underlain by thin loess.	1988–1990	12	1900
	Arsenic sandstone underlain by thin loess.	1988–1990	15	3530
	Arsenic sandstone soil	1988–1990	17	4740
	Bare ground, arsenic sandstone soil	1984–1986	9	3365.1
	Bare ground, loess	1981–1989	17	2285.5
	Bare ground, sandy loess	1983–1984	7	1316
	Bare ground, loess	2012	11.5	4086
	Loess, corn	2012	11.5	2789
	Bare ground, loess	2012	11.5	4296
	Bare ground, loess, corn	2012	11.5	3911
	Bare ground, loess	2013	11.5	12,890
	Bare ground, corn	2013	11.5	9880
	Bare ground, loess	2013	11.5	119.34
	Bare ground, corn	2013	11.5	8010
	Bare ground, loess	2014	11.5	727
	Bare ground, corn	2014	11.5	799
	Bare ground, loess	2014	11.5	523
	Bare ground, corn	2014	11.5	823

### Accuracy validation of soil wind erosion rates

This study referenced wind erosion rate data obtained through various methods within the study area (Table 6) to validate the accuracy and reliability of the results. On flat farmland, wind erosion rates ranged from 59 to 280 t·km⁻²·a⁻¹, with mean values of 216, 252, and 280 t·km⁻²·a⁻¹ in Datong, Lanzhou, and Shenmu, respectively. The overall mean was 249 t·km⁻²·a⁻¹, with a low coefficient of variation, indicating consistency across the region. These results closely align with the wind erosion modulus of 128–197 t·km⁻²·a⁻¹ measured using BSNE sand collectors in the loess areas of Jungar Banner^123^. Moreover, the results are consistent with windward slope (583 t·km⁻²·a⁻¹) and leeward slope (42 t·km⁻²·a⁻¹) data measured using the^7^Be method in Liudaogou, Shenmu^129^, further confirming the reliability of the findings.

The wind erosion rate data also revealed patterns corresponding to topographical variations: wind speeds peaked on windward slope shoulders and gradually decreased on leeward slopes. This trend agrees with Liu’s study in Liudaogou, Shenmu, where the wind erosion rate on sloped farmland was measured at 694 t·km⁻²·a⁻¹ using the^7^Be technique^129^. However, compared to the rates of 1455 t·km⁻²·a⁻¹ reported by Sun in loess slope areas^130^ and the range of 1192–2794 t·km⁻²·a⁻¹ measured using the ¹³⁷Cs method^51,97^, the results of this study are relatively lower. These discrepancies may stem from methodological differences, as techniques like ¹³⁷Cs and^7^Be are more sensitive to specific erosion processes, while BSNE sand collectors are better suited for measuring long-term average rates. Additionally, variations in local topography, vegetation cover, and the temporal scope of the studies may contribute to the observed differences.

To further validate the reliability of the data, comparisons were made with studies conducted in regions with similar climatic conditions abroad. Observations by Sharratt^132^ and Feng^41^ in farmland on the Columbia Plateau in the United States reported wind erosion rates of 112 t·km⁻²·a⁻¹ and 56 t·km⁻²·a⁻¹ for 2003–2004, respectively^88^. These values are generally comparable to the findings of this study, suggesting that wind erosion rates are consistent with those reported in international studies. The relatively lower wind erosion rates abroad may reflect differences in tillage practices, surface cover, and the regulatory effects of climate variability on erosion processes.

In the study of grassland wind erosion rates, the observed range of 145–303 t·km⁻²·a⁻¹ in this study aligns closely with the range of 117–351 t·km⁻²·a⁻¹ reported by Qi and Liu^101,132^. Additionally, wind tunnel experiments conducted by Li reported a wind erosion rate of 115 t·km⁻²·a⁻¹ for grasslands^133^, which is consistent with this study’s results. Such agreement further validates the reliability of the data and demonstrates the relative stability of grassland wind erosion rates within a specific range.

It is worth noting that the wind erosion modulus measured in this study represents multi-year averages for the study area, influenced by factors such as wind speed, vegetation cover, and land use type. In recent years, the implementation of the Grain-for-Green (reforestation) program has significantly improved vegetation cover in the study area, leading to a reduction in soil wind erosion intensity^134^^–^^136^. Additionally, the impact of climate change warrants attention. Studies have shown a trend toward warming and drying in the Loess Plateau, accompanied by a decline in average wind speeds, which has mitigated the frequency and intensity of wind erosion to some extent^137^.

Looking ahead, with the continued advancement of ecological restoration projects and the ongoing effects of climate change, wind erosion rates in the study area are expected to decline further. However, the potential for extreme weather events, such as strong winds and prolonged droughts, remains a concern. Therefore, long-term monitoring and dynamic assessments of wind erosion rates under extreme conditions are essential to ensure the applicability of the research conclusions and to provide scientific guidance for regional soil resource management.

**Table 6:** Measured wind erosion modulus in the agro-pastoral ecotone of Northern China.

Study area	Methods	Wind erosion modulus/average (t km^− 2^a^− 1^)	Land use
Loess plateau (Jungar Banner)	BSNE/power functions	275	Cropland
	BSNE/power functions	213	Cropland
	BSNE/power functions	102	Cropland
	BSNE/exponential function	177	Cropland
	BSNE/exponential function	146	Cropland
	BSNE/exponential function	62	Cropland
Loess plateau (Shenmu county)	^7^Be	78.5	Sloping cropland/leeward slope
	^7^Be	4.7	Slope cultivation/leeward slopes
	^7^Be	778.2	Slope cultivation/windward slope
	^7^Be	388.3	Slope cultivation/windward slope
	^7^Be	1400.2-1849.8/1560	Slope cultivation on sandy loam soil
	^7^Be	336.3-1180.6/694	Cultivated land on clay loam slope
	^137^Cs	1455	Cultivated land
Agricultural and Pastoral Intertwined Zone in Northern China	^137^Cs	1442–2911/2201	Flat cropland
Agricultural and Pastoral Intertwined Zone in Northern China	^137^Cs	1192–2794/1993	fallow land
Republican Basin of Qinghai	^137^Cs	1179–2236/1707	Cropland
Typical Steppe Zone of the Northern Mongolian Plateau	^137^Cs	65 ~ 169/117	Grassland
Jawljit, Mongolia	^137^Cs	280	Grassland desert
Lus, Mongolia	^137^Cs	205	Desertified grassland
Qinghai Republic Basin	^137^Cs	318–1686/1002	Grassland
Inner Mongolia Zhengxiangbai Banner	^137^Cs	351	Grassland
Inner Mongolia Desert Grassland	Wind tunnel experiment	115	Grassland

### Contribution rates of wind and water erosion in the agricultural and pastoral intertwined zone in Northern China

In this study, we determined that the wind-to-water erosion rate ratio in croplands within the agro-pastoral ecotone of northern China is approximately 1:8 to 1:27. This result aligns closely with findings by Yue^60^ in the Shenmu and Liudaogou watershed, where wind and water erosion ratios range from 4:6 to 7:93. However, our ratio is slightly lower than the 1:4.69 reported by Zhang^100^ for the same watershed and the 1:5 ratio calculated by Lin^33^ using alternative models. Moreover, our findings are significantly lower than the 1.8:1 ratio estimated for the Ba Dong River valley sub-region, based on field observations, runoff plots, topographic measurements, and “3S” (GIS, GPS, and RS) technology. These discrepancies are primarily attributed to differences in wind erosion rates reported across studies.

In our research, the wind erosion rate in croplands ranged from 59 to 280 t·km⁻²·a⁻¹, with an average of 190 t·km⁻²·a⁻¹, which is consistent with the 120 t·km⁻²·a⁻¹ measured by Guo^138^ in Zhungeer Banner using a BSNE sand trap. As a widely recognized tool for wind erosion monitoring, the BSNE sand trap has been extensively applied and validated globally, demonstrating high accuracy and reliability. The agreement between our results and those of Guo¹³⁹ further supports the robustness of our findings.

However, our wind erosion rate is considerably lower than the 2100 t·km⁻²·a⁻¹ reported by Yue^60^ using the ¹³⁷Cs method in the Shenmu and Liudaogou watershed and the 1580 t·km⁻²·a⁻¹ obtained by Liu^129^ using the ⁷Be method in the same area. These variations likely arise from differences in measurement methods and techniques, as systematic errors can be introduced by varying methodologies^64^. Additionally, discrepancies in wind erosion rates may be influenced by local climatic conditions, geomorphic characteristics, and wind speed fluctuations during measurement periods, contributing to the observed variations^60,139^.

In contrast, differences in water erosion rates across studies are relatively smaller, typically ranging from 3000 to 8000 t·km⁻²·a⁻¹, indicating the dominant role of water erosion in the study area. This significant disparity underscores the greater influence of water erosion drivers on cropland soil loss in the agro-pastoral ecotone. Regional analyses suggest that the wind-to-water erosion rate ratio generally ranges from 1:3 to 1:25, reflecting strong associations with regional topography, precipitation patterns, and wind regimes. For instance, slope gradient significantly affects water erosion and to a lesser extent influences wind erosion. In areas with intense precipitation, steep slopes can exacerbate water erosion risks, while in regions where slope aspect aligns with wind direction, wind erosion may be amplified.

Further examination of previous studies highlights the critical role of environmental heterogeneity in regulating erosion contribution rates. For example, Yang reported that soil erosion rates increase markedly with slope gradient, with a positive correlation between erosion contribution rates and slope, emphasizing the dominant role of topography in water erosion processes^140^. Similarly, research by D. Tuo^141^ demonstrated that wind force differences along slope aspects significantly influence wind erosion rates. Furthermore, Zhang found that the combined implementation of engineering measures (e.g., retaining walls and diversion ditches) and biological measures (e.g., vegetation cover) exhibits significant variations in regulating erosion contribution rates under different slope conditions^142^. Their findings suggest that in steep-slope areas, relying solely on one type of measure may be insufficient to mitigate erosion effectively, necessitating a multi-factorial and synergistic approach^142^.

Therefore, in northern China’s agro-pastoral ecotone, soil conservation strategies should be tailored to the region’s specific topographic and climatic conditions. For example, in steep-slope areas, a combination of diversion ditches and vegetation buffer zones should be prioritized, whereas in wind-exposed slope-aspect regions, measures such as enhanced vegetation cover and windbreak construction should be emphasized. These strategies can effectively reduce erosion risks, conserve soil resources, and ultimately achieve the goals of ecological restoration and sustainable cropland utilization.

### Methodology of wind-water coupled erosion research

The coupled process of wind and water erosion exhibits certain complexities^143,144^, making it challenging to accurately differentiate the contributions of wind and water erosion^145^. To address this issue, many researchers, both domestically and internationally, have studied wind and water erosion as independent processes in the context of composite erosion research^33,59,60,146^. This study adopts a similar approach, analyzing the relative contributions of wind and water erosion using ¹³⁷Cs tracer technology combined with topographic conditions under reasonable assumptions.

Currently, there are various methods for evaluating the relative contributions of water and wind erosion rates, each with distinct characteristics. Some studies have utilized the Chinese Soil Loss Equation (CSLE) and the Revised Wind Erosion Equation (RWEQ), or the Wind Erosion Model (WEMO) and the Rangeland Hydrology and Erosion Model (RHEM) in New Mexico, USA^33,146^. Additionally, certain studies have integrated simulation models with tracer element measurements, such as those combining the Universal Soil Loss Equation (USLE) with the ¹³⁷Cs tracer method^59,60^. However, wind erosion models (e.g., RWEQ and USLE) exhibit limitations in estimating soil erosion rates, especially in complex terrains and under extreme climatic conditions. The accuracy of these models is highly dependent on precise input parameters, such as wind speed data, which are critical in the RWEQ model^147^. However, spatial variability and temporal fluctuations in wind speed data can significantly impact model predictions, increasing the uncertainty of the results^148,149^.

In addition, some scholars have calculated the erosion energy of wind and water to determine their contributions through fieldwork, observation, and laboratory data analysis^22^. Direct observation methods are also widely used, such as the use of wind erosion material samplers like BSNE and MWAC for wind erosion monitoring, and the use of runoff plot methods and flumes for water erosion sampling^82,150^. Different methods have their own advantages and disadvantages. From the perspective of kinetic energy, wind and water erosion contributions can be easily calculated. However, this approach may overlook the differences caused by microtopographic conditions and subsurface soil properties. Field measurements have the advantage of accurately controlling the source area boundaries of water-eroded sediments, but determining the source area of wind-eroded materials during wind transport remains challenging, which affects the accuracy of wind erosion estimates per unit area^138,151^.

The tracer method is a relatively mature and reliable approach, but it requires careful determination of background values. It is important to note that water erosion models are often validated by extensive runoff plot data, with parameters determined at precise rates. Similarly, wind erosion models have been applied to larger regional assessments. However, when summarizing the wind erosion modeling studies near the study area (Table 7), significant variability in results was observed. In adjacent northern agricultural and pastoral areas, wind erosion modulus varied widely, with maximum values reaching 12,813 t·km⁻²·a⁻¹ and minimum values approaching zero. At the 95% confidence level, wind erosion modulus ranged between 1,959 and 4,657 t·km⁻²·a⁻¹, with an average of 3,190 t·km⁻²·a⁻¹, which was significantly higher than results obtained using tracer methods and direct sand collector measurements. This average was approximately one order of magnitude higher than the tracer and sand collector measurement methods. Thus, we believe that estimating wind erosion modulus using wind erosion models requires high-accuracy base data to ensure reliable results. Otherwise, such models should be used with caution.

In conclusion, future research on wind-water coupled erosion should select appropriate methods based on the characteristics of the study area and research objectives, focusing on improving model applicability and accuracy. Integration of multi-source data, such as combining remote sensing with field measurements, should be emphasized. By optimizing model parameters and improving the quality of input data through the integration of diverse techniques and datasets, researchers can better measure and evaluate wind and water erosion rates^152,153^.

**Table 7:** Statistics of wind erosion modulus estimated by the wind erosion model for the study area and surrounding areas.

Research area	Methods	Wind erosion modulus (t km^− 2^a^− 1^)	Research period
Mongolian plateau	RWEQ	4650	1982–2008
Agricultural and pastoral intertwined region of Northern China	RWEQ	918–5488	2001–2011
Agricultural and pastoral intertwined areas in Northern China	WEPS	1333–12,813	2001–2011
Inner Mongolia Yinshan Northern foothills Region	RWEQ	954–4073	1990–2015
Central and Western inner Mongolia	RWEQ	2219–5234	1990–2015
Inner Mongolia Zhengxiangbai banner	Zhang’s Model	621	2000–2012
Inner Mongolia	RWEQ	2309–5362	1982–2015
Inner Mongolia	RWEQ	3531–6271	2010–2018
Mongolia	WEQ	270–2750	
Yellow River Basin	RWEQ	0-2500	2000–2018
Yellow River Basin	WEPS	556–835	2010–2018
The eastern part of the Qaidam Basin on the Tibetan Plateau.	Wind Erosion Model in China	136	

### Mechanism of wind-water composite erosion

The mechanism of wind-water composite erosion is a complex dynamic process driven by the combined effects of spatiotemporal conditions, geomorphic features, soil properties, and climatic factors. Wind and water erosion do not occur independently; instead, they interact through a series of physical, chemical, and biological mechanisms to form an intricate erosion system.

Wind erosion removes fine surface particles, altering microtopographic features such as the formation of rills or dunes on slopes. These geomorphic changes modify runoff pathways, intensify hydrodynamic concentration effects, and significantly increase water erosion intensity^52,59,154^. Additionally, the removal of surface particles by wind enhances surface roughness and reduces soil infiltration capacity, indirectly increasing the potential for rainfall-induced erosion. At the same time, water erosion exerts feedback effects on wind erosion. For instance, rill channels created by rainfall not only provide pathways for wind-transported materials but also expose soil particles that are more susceptible to erosion^155,156^. Studies have shown that when wind speeds reach 11–14 m s^− 1^, surface roughness and runoff rates increase significantly, leading to a substantial rise in erosion intensity^59,155^.

Wind-water composite erosion exhibits distinct seasonal characteristics. During winter and spring (March to May), strong winds transport fine particles to low-lying areas or the base of slopes, providing abundant material for intense rainfall-induced erosion in summer. In contrast, during summer and autumn (June to August), heavy rainfall redistributes wind-eroded deposits to lower slopes or riverbeds, exacerbating sediment deposition and soil loss. This seasonal alternation is particularly evident in arid and semi-arid regions, where the combination of wind transport and water redistribution makes the erosion system even more complex^22,23,157^.

Spatially, the intensity and patterns of wind-water composite erosion vary significantly due to differences in regional geographic conditions and climatic patterns. For instance, in the black soil regions of northeast China, strong winds in spring and late autumn result in significant wind erosion, while summer rainfall further intensifies water erosion^155^. In the sandy slopes of the Yellow River Basin, erosion rates under intense rainfall are significantly higher than on slopes without sand cover, highlighting the heightened sensitivity of sandy soils to wind-water interactions. Furthermore, freeze-thaw cycles in high-latitude regions exacerbate wind-water composite erosion by destabilizing soil structure, thereby creating more erosion-prone surfaces^155,157^.

From a systemic perspective, wind-water composite erosion is not merely the additive effect of wind and water erosion but a dynamically coupled erosion mechanism. Its driving factors include complex variables such as rainfall intensity, wind speed, surface roughness, and geomorphic characteristics. Extreme weather events, such as heavy rainfall and prolonged droughts, significantly enhance the interactions between wind and water erosion, accelerating soil degradation^24,146^. With the intensification of global climate change, the increased regional frequency of extreme rainfall events and high wind speeds further alters the dynamic patterns of wind-water composite erosion, adding to its complexity.

To gain deeper insights into the mechanism of wind-water composite erosion, future research should integrate high-resolution remote sensing technologies, precise statistical analyses, and laboratory-based simulations to reveal the spatiotemporal dynamics of the erosion system. Additionally, combining field measurement data from various regions will help optimize model parameters and explore the universal patterns and specificities of wind-water composite erosion at regional and global scales. Such efforts not only deepen theoretical understanding but also provide scientific foundations for developing effective soil conservation measures and ecosystem management strategies.

### Shortcomings of this study

This study has achieved certain results; however, several limitations remain that require further exploration and refinement. First, regarding the assumptions made, the study assumes that wind erosion occurs exclusively on flat terrain while both wind and water erosion occur on slopes, with a uniform wind erosion environment. In reality, this assumption may oversimplify the complexity. Wind erosion intensity on flat areas is influenced by various factors such as wind speed, local microtopography, vegetation cover, and soil moisture, which may cause wind erosion to vary rather than occur in a single mode. Similarly, the interaction between wind and water erosion on slopes may exhibit considerable spatial heterogeneity, and assuming uniform occurrence may overlook differences across the slope surface. Future research should thus focus on the spatial variability of wind-water composite erosion under diverse topographical and environmental conditions.

Secondly, while the ^136^Cs tracer method is widely used in soil erosion studies, the limited sample size in this study may affect the representativeness and reliability of the results. The distribution and activity of ^136^Cs are influenced significantly by spatial heterogeneity at sampling sites, and an insufficient sample size may limit a comprehensive capture of the overall trends in wind-water composite erosion. Therefore, increasing the number of sampling points and sample size would improve the accuracy and spatial representativeness of the results, especially over large areas, to better reflect the dynamic changes of wind-water composite erosion.

Additionally, this study does not delve deeply into the specific mechanisms underlying wind-water composite erosion. While the observed interactions between wind and water erosion demonstrate significant impacts in certain areas, their internal mechanisms remain unexplored. For instance, how wind erosion alters surface roughness and soil structure to influence water erosion intensity, or how water erosion exacerbates wind erosion through runoff processes, are complex feedback mechanisms that have not yet been fully examined. Future research should focus on the intricate details of wind-water composite erosion mechanisms, especially across micro, local, and macro scales, to further understand the comprehensive impact of alternating wind and water interactions on soil erosion.

## Conclusions

This study provides critical insights into the mechanisms and impacts of wind and water erosion within the mixed agricultural and pastoral regions of northern China. By employing the ^136^Cs tracer technique, we were able to quantify erosion rates specific to land use types and evaluate the relative contributions of wind and water erosion. The findings reveal that cropland experiences the highest levels of erosion, reaching moderate to severe intensity, while grassland and forested areas are comparatively less affected. These outcomes underscore the need for targeted soil conservation practices, particularly in cropland areas where erosion control measures are most urgently needed.

Our results suggest that water erosion predominates as the main contributor to soil loss in this region. This has important implications for developing erosion control strategies: practices such as slope stabilization, water retention infrastructure, and adaptive land management policies are critical for reducing the erosive impact of rainfall, particularly on sloped croplands. Additionally, sustainable agricultural practices, including reduced tillage and vegetation cover management, should be prioritized to improve soil stability.

Despite the strengths of this study, limitations are present that warrant further investigation. The current methodology assumes uniform wind erosion across high plains and mixed erosion on slopes, potentially overlooking spatial variability due to microtopography and vegetation heterogeneity. Future studies should incorporate these variables more precisely to refine erosion rate estimates. Additionally, expanding the sampling network for ^136^Cs data would enhance the spatial accuracy and robustness of findings, enabling more detailed regional assessments.

Future research should also focus on the interactions between wind and water erosion at a finer scale. Understanding how wind erosion modifies soil surface roughness and influences subsequent water infiltration and runoff could yield deeper insights into erosion dynamics. Further advancements in observational methods, such as integrating ^136^Cs data with high-resolution remote sensing or geospatial analysis, could enable continuous monitoring over larger landscapes. By addressing these aspects, future studies will support more effective soil conservation measures and contribute to sustainable land management across erosion-prone areas of northern China.

## Data Availability

The authors confirm that the data supporting the findings of this study are available within the article.
